# Genetic Evidence for O-Specific Antigen as Receptor of *Pseudomonas aeruginosa* Phage K8 and Its Genomic Analysis

**DOI:** 10.3389/fmicb.2016.00252

**Published:** 2016-03-02

**Authors:** Xuewei Pan, Xiaoli Cui, Fenjiao Zhang, Yang He, Lingyan Li, Hongjiang Yang

**Affiliations:** Key Laboratory of Industrial Microbiology, Ministry of Education, Tianjin Key Laboratory of Industrial Microbiology, College of Biotechnology, Tianjin University of Science and TechnologyTianjin, China

**Keywords:** *Pseudomonas* phage K8, phage receptor, O-specific antigen (OSA), *wzy* gene, genome annotation

## Abstract

Phage therapy requires the comprehensive understanding of the mechanisms underlying the host-phage interactions. In this work, to identify the genes related to *Pseudomonas aeruginosa* phage K8 receptor synthesis, 16 phage-resistant mutants were selected from a Tn*5G* transposon mutant library of strain PAK. The disrupted genetic loci were identified and they were related to O-specific antigen (OSA) synthesis, including gene *wbpR, ssg, wbpV, wbpO*, and *Y880_RS05480*, which encoded a putative O-antigen polymerase Wzy. The Lipopolysaccharide profile of the *Y880_RS05480* mutant was analyzed and shown to lack the O-antigen. Therefore, the data from characterization of Y880_RS05480 by TMHMM and SDS-PAGE silver staining analysis suggest that this locus might encode Wzy. The complete phage K8 genome was characterized as 93879 bp in length and contained identical 1188-bp terminal direct repeats. Comparative genomic analysis showed that phage K8 was highly homologous to members of the genus PaP1-like phages. On the basis of our genetic findings, OSA of *P. aeruginosa* PAK is proven to be the receptor of phage K8. The highly conserved structural proteins among the genetic closely related phages suggest that they may recognize the same receptor.

## Introduction

*Pseudomonas aeruginosa* is an opportunistic pathogen present in diverse environmental niches. It is also one of the most common causes of healthcare-associated infections including pneumonia, bloodstream infections, urinary tract infections, and surgical site infections, accounting for about 9% of all nosocomial infections (Emori and Gaynes, 1993; Lister et al., 2009). Antibiotics are widely used for prevention and control of the infections caused by *P. aeruginosa*, leading to the emergence and the increasing prevalence of multidrug-resistant *P. aeruginosa* clinical isolates (Karaiskos and Giamarellou, 2014). There is an urgent need to discover and develop new classes of antibiotics and alternatives to the conventional drugs. Among the potential candidate antibacterials, lytic phages can kill bacteria efficiently and specifically, bringing great promises to combat drug-resistant pathogens (Kutter et al., 2010; Chan et al., 2013).

Phages are the most abundant biological entities present in various environmental habitats of the Earth’s biosphere, providing a reliable unlimited resource for possible phage applications (Srinivasiah et al., 2008). To date, 1630 phage genomes have been sequenced^1^. One hundred fifty were found to be *P. aeruginosa* phages, including 47 *Myoviridae* phages, 42 *Podoviridae* phages, 46 *Siphoviridae* phages, *Levivirus Pseudomonas* phage PP7 and PRR1 (Olsthoorn et al., 1995; Ruokoranta et al., 2006), *Inovirus Pseudomonas* phage Pf1 (Holland et al., 2006) and Pf3 (Luiten et al., 1985), and 11 uncharacterized dsDNA phage such as PA11 (Kwan et al., 2006) and vB_PaeP_Tr60_Ab31 (Latino et al., 2014). *P. aeruginosa* phages of each family can be further grouped into genus level. *Podoviridae* phages mainly consist of N4-like phages, phiKMV-like phages, T7-like phages, and LUZ24-like phages; *Siphoviridae* phages have four major genera, including D3112-like phages, D3-like phages, Yu-like phages, and Mu-like phages; *Myoviridae* phages are classified into five genera such as phiKZ-like phages, PaP1-like phages, P2-like phages, PB1-like phages, and KPP10-like phages^1^.

Though great progress has been made in phage discovery and applications, the underlying mechanisms of host–phage interactions still remain to be elucidated. In this study, *P. aeruginosa* phage K8 was selected for biological characteristic analysis, phage genome annotation, and screening for host genes encoding the phage receptors.

## Materials and Methods

### Bacterial Strains, Phage, Plasmids, and Growth Conditions

Phage K8 was first isolated using *P. aeruginosa* PAK as the indicator strain from Haihe river located in Tianjin, China (Li et al., 2010). Bacterial strains were grown in LB medium at 37°C. When appropriate, the medium was supplemented with ampicillin (100 μg/ml) or gentamicin (10 μg/ml) for *Escherichia coli* strains cultivation; and carbenicillin (150 μg/ml) or gentamicin (100 μg/ml) for *P. aeruginosa* strains cultivation. *P. aeruginosa* SK2, SK5, SK15, SK16, SK21, SK23, SK24, SK28, SK41, SK45, SK73, SK75, SK88, SK91, SK92, and SK98 were phage K8-resistant mutants derived from the Tn*5G* transposon mutagenesis bank of strain PAK. Vector pUCP18 was used to express the target genes in the isolated phage-resistant mutants for complementation tests (**Table 1**).

**Table 1:** Strains, phage, and plasmids used in this study.

Strain, phage or plasmid	Description	Source
***Pseudomonas aeruginosa* strains**		
PAK	Laboratory strain, serotype O6	Bradley and Khan, 1974
SK28, SK45	*wbpR::Gm^R^* mutant of PAK, resistant to phage K8	This study
SK2, SK16, SK23, SK91	*wbpV::Gm^R^* mutant of PAK, resistant to phage K8	This study
SK5, SK15	*wbpO::Gm^R^* mutant of PAK, resistant to phage K8	This study
SK98	*ssg::Gm^R^* mutant of PAK, resistant to phage K8	This study
SK21, SK41, SK73, SK75, SK88, SK92, SK24	*Y880_RS05480 ::Gm^R^* mutant of PAK, resistant to phage K8	This study
***Escherichia coli* strain**		
DH5α	*hsdR recA lacZYAΦ80 lacZΔM15*	BRL
**Phage**		
K8	Lytic bacteriophage specific to PAK strain	Li et al., 2010
**Plasmids**		
pRK2013Tn*5G*	Tn*5G* carrying plasmid, Km^R^Gm^R^	Nunn and Lory, 1992
pGEM-T Easy	Cloning vector for the PCR products, Ap^R^	Promega
pUCP18	Broad-host-range shuttle vector, Ap^R^	Schweizer, 1991
pXL1503	*wbpR* gene with its own promoter cloned in pUCP18, Ap^R^	This study
pLY1201	*Y880_RS05480* gene with its own promoter cloned in pUCP18, Ap^R^	This study
pFJ1501	*wbpV* gene with its own promoter cloned in pUCP18, Ap^R^	This study
pXW1501	*ssg* gene driven by *P_lac_* cloned in pUCP18, Ap^R^	This study
pXW1503	*wbpO* gene driven by *P_lac_* cloned in pUCP18, Ap^R^	This study
pXW1504	*wbpP* gene driven by *P_lac_* cloned in pUCP18, Ap^R^	This study
pXW1505	*wbpOP* gene with its own promoter cloned in pUCP18, Ap^R^	This study

### Transmission Electron Microscopy (TEM)

Phage particles were purified as described previously (Yang et al., 2010). In brief, phage K8 lysate (about 10^11^ pfu/ml) was treated with DNase I (5 μg/ml) and RNase A (5 μg/ml) at 37°C for 1 h. With the addition of 0.1 M NaCl, the mixture was kept on ice bath for 1 h and spun at 12000 × *g* for 20 min. The collected supernatant was supplemented with PEG6000 (10%) and stored at 4°C overnight before centrifuging at 12000 × *g* for 20 min. Phage pellet was suspended with 2% ammonium acetate (pH 7.0) and filtrated with Amicon-100 filter. The purified phages were adsorbed onto a carbon-coated copper grid for 5 min, and subsequently negatively stained with 2% phosphotungstic acid (pH 6.7) for 5 min. Morphology observation was carried out with a JEM-1400 transmission electron microscope operating at 100 kV.

### Latent Period and Burst Size Analysis

Latent period and burst size of phage K8 was determined by one-step growth experiment described previously (Yang et al., 2010). Briefly, PAK cells were harvested from the 50 ml culture (OD_600_ at 0.6) and suspended in 0.5 ml LB medium. The suspension was mixed with 0.5 ml appropriately diluted phage K8 solution at a MOI (multiplicity of infection) of 0.0001. After adsorption for 1 min, the mixture was spun at 13000 × *g* for 30 s to remove free phage particles. The pellet was resuspended in 100 ml LB medium for immediate cultivation. At 5 min intervals, samples were taken and the infection centers were determined by the double-layer agar plate method (Li et al., 2010).

### Screening of Phage Resistant Mutants

Tn*5G* transposon was used to mutagenize *P. aeruginosa* PAK to construct an insertional mutant library as described earlier (Nunn and Lory, 1992). After mating, a fraction of the mutant bank was mixed with the stock solution of phage K8 and incubated for 4 h with a shaking speed of 220 rpm. Aliquots were plated onto L-agar medium with 100 μg/ml gentamicin and 100 μg/ml ampicillin. The grown colonies were selected as the phage-resistant mutants. The mutants were confirmed by the spotting assay and the double-layer plate method as described previously (Li et al., 2010). The adsorption rate of the phage-resistant mutants was determined as described previously (Yang et al., 2010).

### Identification of the Transposon Insertion Sites by Inverse PCR

Inverse PCR was performed as described previously (Wang et al., 1996). In brief, chromosomal DNA was isolated from the phage-resistant mutants, digested with the restriction enzyme *Taq*I or *Pst*I, self-ligated, and amplified using primers OTn1 and OTn2, Tn1 and Tn2, or F1 and R1, respectively (**Table 2**). The PCR products was sequenced directly or cloned into pGEM-T Easy vector for sequencing. The obtained sequences were analyzed by searching the genome database of *Pseudomonas* strains^2^.

**Table 2:** Primer used in this study.

Primer	Sequence (5′–3′)	Function
OTn1	GATCCTGGAAAACGGGAAAG	Identification of Tn5G in mutants
OTn2	CCATCTCATCAGAGGGTAGT	
Tn1	AGCGCCGCCGAAGAGAACAC	Identification of Tn5G in mutants
Tn2	GGCTGGCGCCATGCAAACAG	
F1	CCCGCGGATGGTGGGTTCAC	Identification of Tn5G in mutants
R1	GCGACGTTAACCAAGCGGGC	
SSG-F	CGCAAGCTTTCTTCATCGGTCCTACAC	Amplification of gene *ssg*
SSG-R	CGCAAGCTTAGTTGTTCTGGGTGGAGT	
05480 -F	CGCAAGCTTCCGGGCTTCCAGCTCCTGGATCTTTTG	Amplification of gene *Y880_RS05480*
05480 -R	CGCAAGCTTCAACGCAGAACGACGGAAGTTTGGCAC	
WbpV-F	CCCAAGCTTCCAGCAGGAAGGAGAGCACG	Amplification of gene *wbpV*
WbpV-R	CCCAAGCTTGTGCCTGTGTCGCCTGGCTTTA	
WbpR-F	CGCGGATCCAGAACACCGACGCCCTGG	Amplification of gene *wbpR*
WbpR-R	CGCGGATCCCAACAAGCCGCTGAAGCC	
WbpO-F	CGCGGATCCAATCAGCCAGACTTTCGG	Amplification of gene *wbpO*
WbpO-R	CGCGGATCCTAGGGTCGGCAGAAGTTT	
WbpP-F	CGCTCTAGATACTTTCATCCAAACGCA	Amplification of gene *wbpP*
WbpP-R	CGCTCTAGATGGCGGAATACAACATAC	
WbpOP-F	CGCGAGCTCGCACCAGGCGACTCTCAAA	Amplification of gene *wbpOP*
WbpOP-R	CGCGAGCTCGTGAGAGGTGGGTTTAGGCG	
M-1	TCGCTCTTTTCTACGGGACA	Identification of 5′ terminus
M-2	GTTCGCCTTCTGCCAGTTAT	
M-3	GACTCCAGCCCAGCAAATAC	Identification of 3′ terminus
M-4	TCTCAGACGATGCCAGTTGT	

The primers were designed to amplify the target genes disrupted in the phage-resistant mutants. The PCR products were subsequently cloned into the multiple cloning sites of plasmid pUCP18. The recombinant plasmids were transformed into the phage-resistant mutants. Sensitivity to phage K8 was tested in the transformants with the spotting and the double-layer method (Li et al., 2010).

### Transmembrane Helices Prediction and Lipopolysaccharide (LPS) Profile Analysis

The transmembrane helices of the putative O-antigen polymerase encoded by gene *Y880_RS05480* was analyzed by the software TMHMM 2.0^3^ (Krogh et al., 2001).

Lipopolysaccharide (LPS) was extracted using the hot water-phenol method as described previously (Westphal and Jann, 1965). In brief, PAK cells in 100 ml culture (OD_600_ at 1.0) were harvested and subjected to the treatments of hot water and phenol sequentially. The residual phenol was removed by dialysis in water. The LPS solution was concentrated by dialysis in 40% PEG6000 solution. DNase I (10 μg/ml) and RNase A (100 μg/ml) were added to remove the residual nucleic acid in the LPS samples. LPS was analyzed by 12% SDS-PAGE and visualized by the silver staining method as described previously (Fomsgaard et al., 1990).

### Biofilm Assay

Biofilm production was assessed in wild-type strain PAK and the phage-resistant mutants as described previously (Brencic et al., 2009). In brief, LB medium was diluted three times and used for biofilm production. The cultivation was carried out in 96 well plates and incubated at 37°C for 48 h. Biofilm was quantitatively measured with the crystal violet staining method (Djordjevic et al., 2002).

### Phage Genome Sequencing and Annotation

Purified phage particles were subjected to genomic DNA extraction according to the method described previously (Yang et al., 2010). Genome sequencing was carried out by Hiseq Illumina 2500 in GENEWIZ, Inc., China^4^. The adaptor sequences were removed with the software Trimmomatic v0.30 (Bolger et al., 2014). A total of 7803122 reads and 773403396 bp were obtained as clean data without any uncertain bases. The sequences were assembled with the software Velvet_v1.12.10 (Zerbino and Birney, 2008). DNA Master was used for the phage genome annotation^5^ by searching against the non-redundant protein database (nr) from NCBI (Wheeler et al., 2003). The software tRNAscan-SE v1.21 was used to predict tRNA genes (Schattner et al., 2005). GC content was determined with the software DNAStar. The GC skew was analyzed by the software DNAPlotter (Carver et al., 2009).

### Identification of Phage Genome Termini

Sequencing depth was analyzed across the assembled genome to find the high-frequency sequences (HFSs) which might represent the phage genome termini (Li et al., 2014). Restriction enzyme cleavage sites and restriction mapping in linear or circular genome sequences were simulated using the software DNA Master. The fragments containing the possible 3′ or 5′ terminus of the K8 genome were purified by the agarose gel electrophoresis. The purified DNA fragments were treated with the Klenow fragment and T4 DNA ligase sequentially. PCR was performed with the specific primers (**Table 2**) and the PCR products were sequenced to identify the genome termini. The assembled genome was curated and the complete genome sequence was deposit in GenBank in NCBI with the accession number KT736033.

### Comparative Genomic Analysis

The homology search of the K8 genome sequence was performed against the NCBI nucleotide database. Four phage genome sequences were selected for comparison analysis by the software Mauve, including PaP1, JG004, PAK_P2, and vB_PaeM_C2-10_Ab1 (Darling et al., 2004). The tail fiber proteins of phage K8 were analyzed and the phylogenetic trees was constructed with MEGA5 (Tamura et al., 2011).

## Results

### Characteristics of Phage K8

**FIGURE 1:**
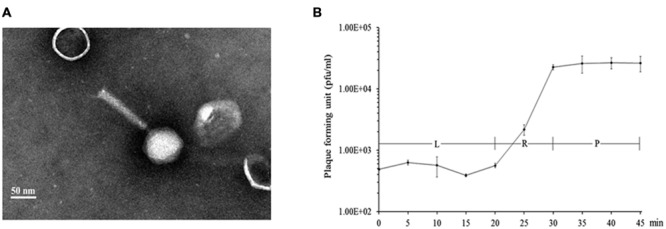
**Characteristics of phage K8. (A)** Electron microscopic image of negatively stained phage K8 particle. The scale bar represents 50 nm. **(B)** One-step growth curve of phage K8. L, latent phase; R, rise phase; P, plateau phase.

Purified phage K8 particles were negatively stained using 2% phosphotungstic acid and observed by TEM. The obtained images showed that phage K8 has an icosahedral head structure connected with a contractile tail. The phage head was about 76.0 nm in diameter and the tail was about 122.0 nm in length (**Figure 1A**). The observed morphology indicated that phage K8 should be tentatively classified as a member of *Myoviridae* family. The progeny production of phage K8 was characterized by one-step growth experiment with a MOI of 0.0001. As inferred from the triphasic curve, the latent period was about 20 min and the burst size was about 46.3 pfu/infection center (**Figure 1B**).

### Identification of Phage Receptor Related Genes

**FIGURE 2:**
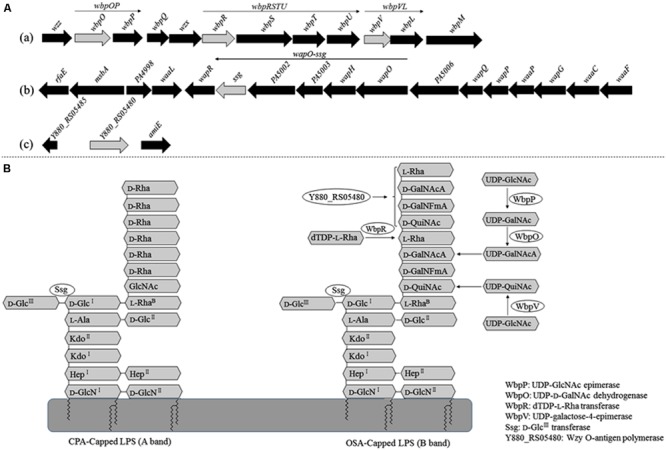
**Analysis of the genes related to the synthesis of phage K8 receptor. (A)** The genetic loci identified in phage resistant mutants. Light gray arrows represent the disrupted genes by Tn*5G* transposon. (a) The *wbp* gene cluster of PAK related to O-specific antigen (OSA) Lipopolysaccharide (LPS) synthesis, including *wbpOP* operon, *wbpVL* operon, and *wbpRUST* operon. (b) The 17 genes cluster of PAO1 involved in core oligosaccharide (OS) moiety biosynthesis, including *wapO*-*ssg* operon. (c) *Y880_RS05480* gene in PAK genome, encoding a Wzy O-antigen α-polymerase. **(B)** The scheme of LPS structure and the enzymes required by LPS biosynthesis.

A random Tn*5G* transposon library of *P. aeruginosa* PAK was constructed to identify the host genes involved in the phage infection process. A total of 16 phage K8 resistant mutants were isolated. With inverse PCR, five different genes were identified disrupted in the mutated strains, including two mutants with the inactivated gene identical to *wbpR* gene of strain LESB58, four mutants with the inactivated gene identical to *wbpV* gene of strain PA96, 1 mutant with the inactivated gene identical to *ssg* gene of strain PAO1 (Veeranagouda et al., 2011), two mutants with the inactivated gene identical to *wbpO* gene of strain PA96, and seven mutants with the inactivated gene *Y880_RS05480* encoding a probable O-antigen polymerase with 22.1% identity to Wzy (AIG62435) of *E. coli* at the amino acid sequence level (**Figure 2** and Supplementary Table S1).

### Adsorption Rate Analysis and Confirmation of the Phage Resistant Mutants

**FIGURE 3:**
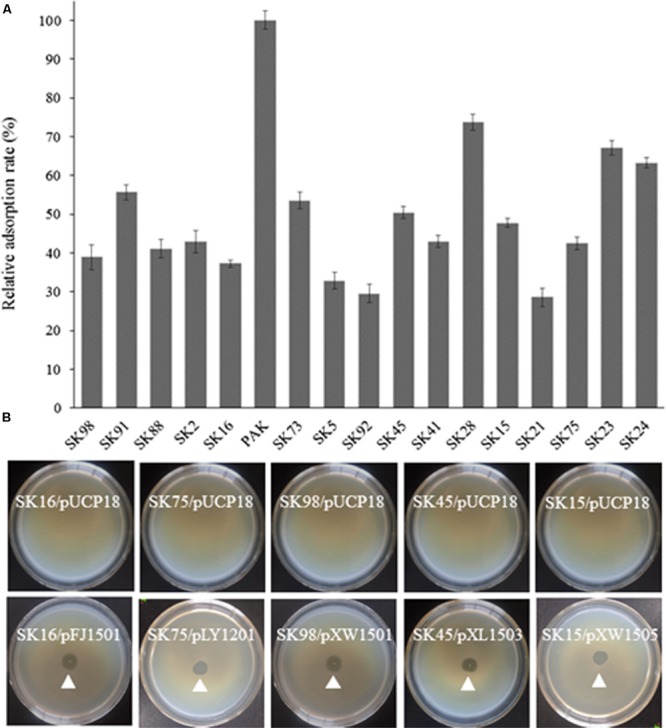
**Characterization of the isolated phage resistant mutants. (A)** The relative adsorption rate of phage resistant mutant. The parent strain PAK is used as control. **(B)** Phage sensitivity test of the isolated mutants. The mutants SK16, SK75, SK98, SK45, and SK15 have disrupted genes *wbpV, Y880_RS05480, ssg, wbpR*, and *wbpO*, respectively. Plasmid pFJ1501 carries *wbpV* gene; pLY1201 carries *Y880_RS05480* gene; pXW1501 carries *ssg* gene; pXL1503 carries *wbpR* gene; and pXW1505 carries *wbpOP* genes.

The adsorption ability of the phage-resistant mutants was analyzed. Compared with the parent strain PAK, the relative adsorption rates of phage K8 to the mutants were between 28.5 and 73.7%, implying that the phage receptors were impaired in these mutants (**Figure 3A**). Complementation test was carried out for each mutant. Gene *wbpR, wbpV, ssg*, and *Y880_RS05480* were cloned and complementation successfully restored the sensitivity to phage K8 in the corresponding mutants, while gene *wbpO* and *wbpP* were both required for the phage sensitivity restoration in the *wbpO* mutant (**Figure 3B**). The results indicated that the disrupted genes in the mutants were responsible for the phage resistance phenotype.

### Function Analysis of Gene Y880_RS05480

**FIGURE 4:**
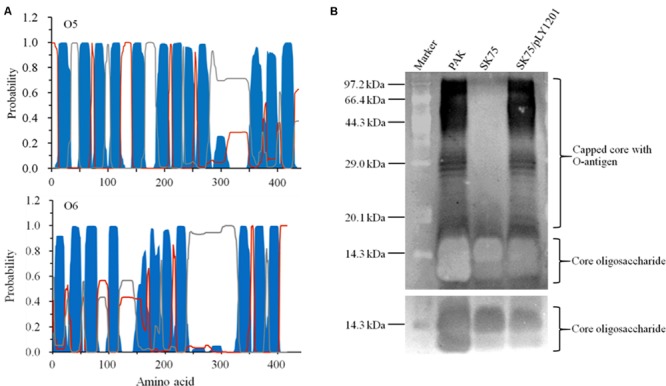
**Characterization of the protein encoded by *Y880_RS05480* gene. (A)** Prediction of the transmembrane structures by TMHMM 2.0. Blue: the transmembrane helices. Gray: the periplasmic loops. Red: the cytoplasmic loops. The upper panel displays the structure of Wzy O-antigen polymerase of the serotype O5 strain PAO1 and the lower panel displays the structure of the hypothetical protein Y880_RS05480 of the serotype O6 strain PAK. **(B)** LPS profiles analysis with the silver-stained SDS-PAGE gel. M: the protein standard marker. PAK: the wild-type strain. SK75: the *Y880_RS05480* mutant. pLY1201: the recombinant plasmid carries *Y880_RS05480* gene. The upper panel displays the long-exposure photography for visualizing the O-antigen, while the lower panel displays the short-exposure photography for visualizing the core oligosaccharide.

Lipopolysaccharide (LPS) is comprised of two forms of O-antigen, the common polysaccharide antigen (CPA) and the O-specific antigen (OSA). Wzy O-antigen polymerases are essential for O-antigen biosynthesis. They exhibit the low sequence conservation among the *P. aeruginosa* strains with the different serotypes. Currently Wzy proteins are not identified in O6, O7, and O8 strains of *P. aeruginosa* despite the fact that they produce normal O-antigen on the surface of their cells (Islam and Lam, 2014). The inactivated gene *Y880_RS05480* encodes a hypothetical protein sharing 22.1% identity to the Wzy O-antigen polymerase (AIG62435) of *E. coli*. With the TMHMM prediction service, this hypothetical protein displayed a large periplasmic loop in its C-terminal and 11 transmembrane helices (**Figure 4A**), similar to the topology of the Wzy proteins found in the *P. aeruginosa* serotype O5 strain PAO1 and the other serotype strains (Islam and Lam, 2014).

The LPS profiles were analyzed in the strains PAK, the *Y880_RS05480* mutant SK75, and the mutant carrying the intact gene *Y880_RS05480* (SK75/pLY1201). The *Y880_RS05480* mutant SK75 was devoid of the O-antigen with high molecular weight, whereas the wild-type strain PAK and the mutant carrying the intact the *Y880_RS05480* gene produced the normal pattern of O-antigen LPS, including O-antigen and core oligosaccharide (**Figure 4B**). The results indicate that the product of gene *Y880_RS05480* may have a similar function in the serotype O6 strain PAK as the Wzy O-antigen polymerases in their corresponding strains.

### Biofilm Production Assay

**FIGURE 5:**
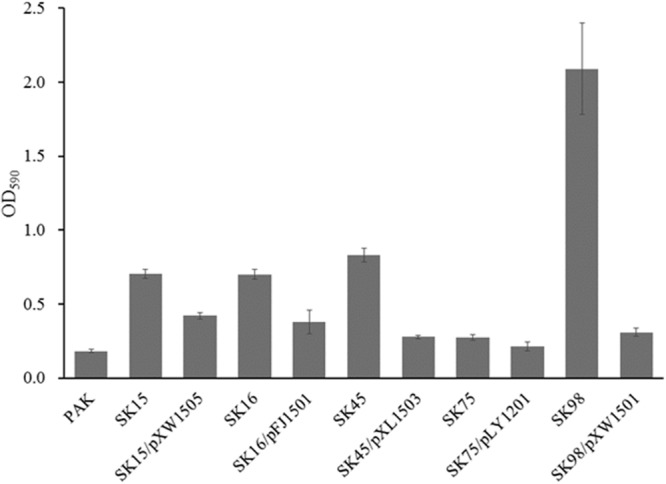
**Biofilm production assay.** The mutants SK16, SK75, SK98, SK45, and SK15 have the disrupted genes *wbpV, wzy* (*Y880_RS05480*), *ssg, wbpR*, and *wbpO*, respectively. Plasmid pFJ1501 carries *wbpV* gene; pLY1201 carries *wzy* (*Y880_RS05480*) gene; pXW1501 carries *ssg* gene; pXL1503 carries *wbpR* gene; and pXW1505 carries *wbpOP* genes.

Strains with different LPS phenotypes produced different amounts of biofilm under various conditions (Murphy et al., 2014; Ruhal et al., 2015). The phage-resistant mutants were analyzed for biofilm production after 48 h incubation. The mutants yielded 1.5–11.5 times biofilm compared with the wild-type strain PAK, and the *ssg* mutant produced the highest level of biofilm (**Figure 5**; Veeranagouda et al., 2011). When the mutants were complemented with their corresponding genes, the resulted strains produced significantly less amount of biofilm (**Figure 5**). The results indirectly indicated the phage-resistant mutants had altered LPS profiles.

### Identification of the K8 Genome Termini

**FIGURE 6:**
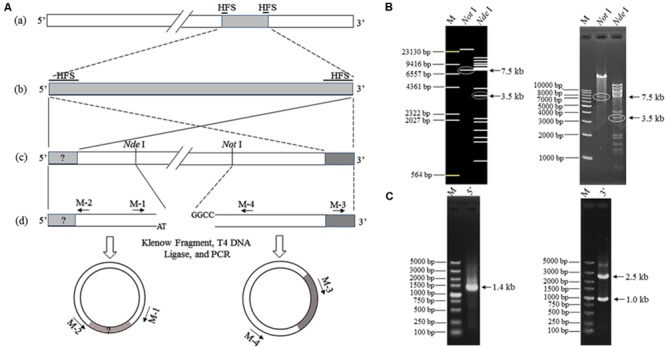
**Termini identification of phage K8 genome. (A)** The scheme for termini identification. (a) The assembled K8 genome of 92769 bp. HFSs refers to high-frequency sequences of 102 bp. (b) The two HFSs and the internal sequence. (c) The curated K8 genome with the proposed termini. Restriction enzymes *Nde*I and *Not*I are selected to digest the K8 genome for obtaining the fragment containing the 5′ or 3′ terminus. (d) Primers M-1, M-2, M-3, and M-4 are used for amplification of the termini. **(B)** Electrophoresis of the K8 genome digested by the restriction enzyme *Nde*I or *Not*I, respectively. The left panel is from the *in silico* simulation and the right panel was from the restriction enzyme digestions. The arrows point at the target fragments. **(C)** The left panel represents the PCR product containing the proposed 5′ terminus; the right panel represents the PCR product containing the proposed 3′ terminus. M: DNA markers.

Two 102-bp HFSs were found with the sequencing depths 62.8 times over the average level in the assembled phage K8 genome, possibly representing the termini of phage genome (Li et al., 2014). Based on this prediction, the restriction mapping of the enzyme *Not*I and *Nde*I was simulated, respectively (**Figures 6A,B**). Enzyme *Not*I digestion produced one specific 7.5-kb fragment containing 3′ terminus (**Figure 6B**). Enzyme *Nde*I digestion produced a 3.5-kb fragment instead of the proposed 2.3-kb fragment, indicating that the 5′ terminus included a piece of unknown DNA fragment of about 1.2-kb was absent from the draft K8 genome (**Figure 6B**). The resultant 7.5 and 3.5-kb fragments were further found including identical 1188-bp sequences, demonstrating that the K8 genome has the identical terminal direct repeats. The 1.0-kb PCR product was also analyzed and was part of the 3′ terminus possibly amplified from the phage genome fragments (**Figure 6C**).

### Genome Structure and Annotation of Phage K8

**FIGURE 7:**
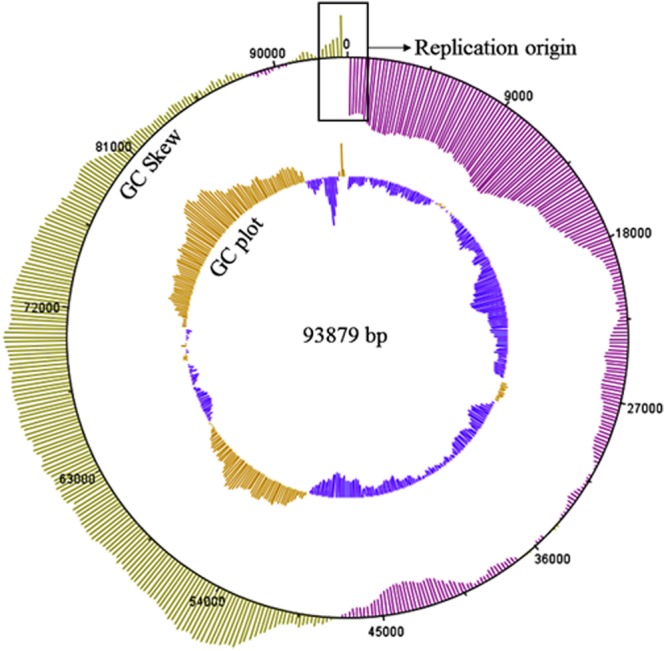
**GC skew and GC plot of the K8 genome.** The outer circle represents the value of the GC skew, green for positive and pink for negative. The inner circle represents the value of GC plot, yellow for G+C content above the average level 49.35% of the K8 genome and blue for G+C content below the average level of the K8 genome. The sequences in the rectangular box stand for the putative replication origin of the K8 genome.

The curated K8 genome has 93879 bp in length. The G+C content of the K8 genome is 49.35%. The abundance ratio of guanine to cytosine was analyzed by GC Plotter. The result showed that an asymmetric nucleotide composition was located near the virtual junction region between the termini of the K8 genome (**Figure 7**). The asymmetry might correspond to the DNA replication origin and the putative replication initiation site of phage K8 genome (Necsulea and Lobry, 2007).

**FIGURE 8:**
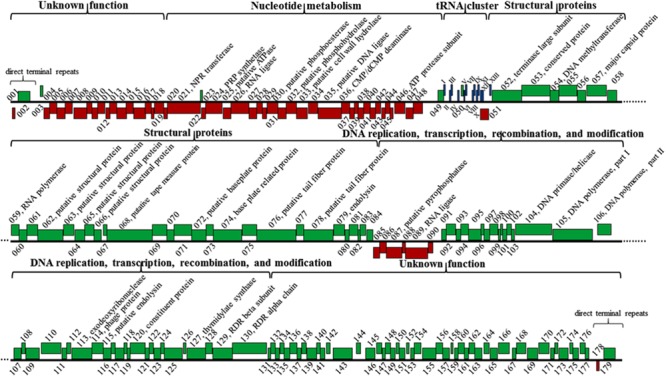
**Genomic structure of phage K8.** Red boxes represent genes on the minus strand. Green boxes represent genes on the plus strand. Roman numerals I-XIII refer to the genes encoding tRNA^Gln^, tRNA^Arg^, tRNA^Lys^, tRNA^Leu^, tRNA^Ile^, tRNA^Asp^, tRNA^Cys^, tRNA^Asn^, tRNA^Pro^, tRNA^Gly^, tRNA^Phe^, tRNA^Glu^, and tRNA^Thr^, respectively. NPR, nicotinamide phosphoribosyl; PRP, phosphoribosyl pyrophosphate; RDR, ribonucleotide diphosphate reductase.

The K8 genome has 179 predicted protein-coding genes distinctively arranged in five major clusters (**Figure 8**). (i) Genes in the cluster I mainly encoded proteins related to nucleotide metabolism, most of them shared great similarities with their homologs except for gene *087* encoding the pyrophosphatase that only shares 43.7% similarity with that of *Burkholderia* phage AH2 (**Figure 8** and Supplementary Table S2). (ii) Cluster II has 10 genes encoding structural proteins and unclassified structural proteins. All proteins shared great similarities of 98.6–100% to their counterparts of phage PaP1 (**Figure 8** and Supplementary Table S2) (Lu et al., 2013). (iii) Cluster III genes mainly encoded proteins related to DNA replication, transcription, recombination, and modification processes (**Figure 8** and Supplementary Table S2). (iv) Genes in the cluster IV and V encoded proteins with unknown functions, and each cluster was adjacent to the termini of the K8 genome, respectively (**Figure 8** and Supplementary Table S2). Thirteen tRNA genes were organized in one minor cluster between cluster I and II (**Figure 8** and Supplementary Table S2).

Three endolysins encoding genes were identified, including the putative cell wall hydrolase (gene *033*) belonging to the hydrolase-2 family located within the cluster I region; the endoylsin (gene *079*) identical to that of phage PaP1 located within the cluster II region; and the putative endolysin (gene *115*) located within cluster III sharing 40.8% identity with that of *Pseudomonas* phage LU11 (Adriaenssens et al., 2012). However, no holin encoding gene was identified in phage K8 genome (**Figure 8** and Supplementary Table S2).

### Comparative Genomic Analysis

**FIGURE 9:**
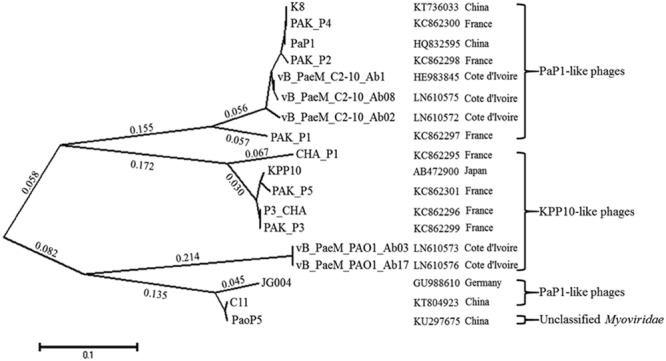
**The phylogenetic tree of the tail fiber proteins with MEGA5.** Seventeen tail fiber proteins which share 47–100% amino acid sequence identity to the putative tail fiber protein (076) of phage K8 are included. *Pseudomonas aeruginosa* phage PaoP5 is unclassified. The geographic locations represent the sites where the phages are isolated.

Homology of the K8 genome sequence was searched in NCBI. The result showed that the K8 genome has high similarities (>90%) and coverage (>90%) with phage PaP1, JG004, PAK_P2, vB_PaeM_C2-10_Ab1, PAK_P4, and PAK_P1. Comparative genomic analysis was further performed with the software Mauve (**Supplementary Figure S1**). Though the K8 genome was highly homologous to the reference genomes, genetic differences were found within the phage group. Compared to the K8 genome, PaP1 has six genes absent in its genome (Lu et al., 2013), JG004 has 10 genes absent in its genome (Garbe et al., 2011), PAK_P2 has 12 genes absent in its genome (Henry et al., 2015), and vB_PaeM_C2-10_Ab1 has 10 genes absent in its genome (Essoh et al., 2013). All absent genes were located within the gene clusters IV and V with unknown functions except for gene *093* which was positioned in middle of the K8 genome (Supplementary Table S2). The tail fiber proteins can act as the ligands to recognize the phage receptors during the infection process. The phylogenetic relationship was investigated among the 18 most homologous tail fiber proteins of *P. aeruginosa* phages including K8. The proteins were grouped into four clades on the basis of homology. The analysis showed that the phylogenetic distance of the tail fiber proteins was not correlated with the geographic locations where the phages were isolated (**Figure 9**).

## Discussion

*Pseudomonas aeruginosa* phages that have been identified so far are comprised of at least 24 genera classified into *Podoviridae, Myoviridae*, and *Siphoviridae* families (Sepulveda-Robles et al., 2012). Phage K8 exhibits an icosahedral head structure with a contractile tail and is classified into the *Myoviridae* family. Its genome is highly homologous to that of phage PaP1 and their major capsid proteins are identical, suggesting that phage K8 is a new member of the PaP1-like phages (Lu et al., 2013) or PAK_P1-like phages genus (Henry et al., 2015). To date, the genus includes 18 phages besides phage K8 (Essoh et al., 2015). Though these phages were isolated from France, Germany, Côte d’Ivoire, Chongqing (China), and Tianjin (China), the phage genomes share great similarities. The result is consistent with the findings that *P. aeruginosa* phages of specific genera are genetically closely related and can be readily isolated from environmental samples globally (Ceyssens and Lavigne, 2010).

The terminal structure of the dsDNA phage genomes has at least five major types, including the linear genomes with 5′-protruded cohesive ends (Tan et al., 2007); the linear genomes with 3′-protruded cohesive ends (Zeigler, 2013); the linear genomes with terminal direct repeats (Pajunen et al., 2001); the genomes with circular permutation and terminal redundancy with specific *pac* recognition sites (Alonso et al., 1997); and the genomes with circular permutation and terminal redundancy without specific *pac* recognition sites (Miller et al., 2003). Many *P. aeruginosa* phages have similar direct terminal repeats with lengths ranging from 184 to 1238 bp, including PaP1-like phages, KPP10-like phages, and some *Podoviridae* phages (Ceyssens et al., 2006; Henry et al., 2015). The direct terminal repeats are highly conserved among the PaP1-like phages genus and may be related to the patterns of viral genome replication in these phages.

Diverse receptors of *P. aeruginosa* phages have been identified. Phage PA1Ø, MPK7, B3, and D3112 use type IV pili as the receptor for infection (Roncero et al., 1990; Kim et al., 2012; Bae and Cho, 2013). Phage phiCTX and H22 use core oligosaccharide of LPS as the receptor (Temple et al., 1986; Yokota et al., 1994). Phage FIZ15 and D3 use LPS O-antigen as the receptor (Kuzio and Kropinski, 1983; Vaca-Pacheco et al., 1999). Phage A7 use CPA as the receptor (Rivera et al., 1992). Phage PIK receptor in LPS contains D-mannose, L-rhamnose, and D-glucosamine and may be the heteropolymer O-antigen OSA (Patel and Rao, 1983). For phage JG004, a series of genes related to LPS pathway have been identified involved in the receptor synthesis (Garbe et al., 2011).

Lipopolysaccharide is described as a molecule with three domains, lipid A, core oligosaccharide, and O-antigen. *P. aeruginosa* PAK simultaneously synthesizes two different forms of O-antigen. CPA is a homopolymer of D-rhamnose (D-Rha). OSA is a heteropolymer composing of repeating units of D-QuiNAc, D-GalNAcA, D-GalNFmA, and L-Rha (Belanger et al., 1999). In this study, all the disrupted genes related to the phage receptor synthesis play a key role in LPS biosynthesis in *P. aeruginosa* PAK. WbpR is a putative dTDP-L-Rha transferase, adding the fourth residue L-Rha to the repeating unit of OSA in O6 strains (**Figure 2**; Belanger et al., 1999). Gene *wbpV* encodes a UDP-galactose-4-epimerase involved in the pathway of UDP-QuiNAc synthesis. UDP-QuiNAc is further added to the repeating unit of OSA as the first residue D-QuiNAc by the glycosyltransferase WbpL (Rocchetta et al., 1999). Gene *wbpP* is located downstream of gene *wbpO* within the same operon, encoding the epimerase converting UDP-GlcNAc to UDP-GalNAc (Creuzenet et al., 2000). Gene *wbpO* encodes the dehydrogenase converting UDP-GalNAc to UDP-GalNAcA. UDP-GalNAcA is further added as the third residue of the repeating unit of OSA (**Figure 2**; Zhao et al., 2000). The cluster of 17 genes of *P. aeruginosa* has been found involved in core oligosaccharide (OS) moiety biosynthesis. Among them, gene *ssg* encodes a glycosyltransferase and is responsible for the transfer of α-D-Glc^III^ to OS moiety (**Figure 2**; Lam et al., 2011; Veeranagouda et al., 2011). Both CPA and OSA are lost in the *ssg* mutant strain (Fernandez et al., 2013). *P. aeruginosa* serotype O6 strains are able to synthesize long-chain O antigen. However, no *wzy* gene homolog is identified within the *wbp* gene cluster for O-antigen synthesis in O6 strains. In this work, though the protein encoded by the gene *Y880_RS05480* displays less similarities with the known Wzy polymerases, the *Y880_RS05480* mutant isn’t able to produce the O-antigen with the high molecular weight, indicating the Wzy-dependent pathway existed in O6 strain PAK for LPS synthesis (Islam and Lam, 2014).

## Conclusion

Five genes *wbpR, wbpV, wbpO, ssg*, and *wzy* are identified as inactivated in the phage-resistant mutants. Gene *Y880_RS05480* is first proved to function as the Wzy O-antigen polymerases. In combination, the results indicate that OSA should be the receptor of phage K8.

## Author Contributions

XP performed the bioinformatic analysis and experiments and wrote the manuscript. XC, FZ, and LL carried out the plasmid constructions. YH performed the bioinformatic analysis. HY designed the experiments and wrote the manuscript.

## Conflict of Interest Statement

The authors declare that the research was conducted in the absence of any commercial or financial relationships that could be construed as a potential conflict of interest.

## Footnotes

1 http://www.ncbi.nlm.nih.gov/genomes

2 www.pseudomonas.com

3 http://www.cbs.dtu.dk/services/TMHMM/

4 http://www.genewiz.com/

5 http://cobamide2.bio.pitt.edu

## Supplementary Material

FIGURE S1 **Comparative genomic analyses of *Pseudomonas aeruginosa* phages.** Ab1: vB_PaeM_C2-10_Ab1. The coordinate rulers display the size of the corresponding genomes. The height of the red and green ribbons is correlated with the level of similarities between every two genomes. Different colors indicate the inconsistent genomic organizations among the phages.
