# Supplementary figures and images for: Genetic Evidence for O-Specific Antigen as Receptor of Pseudomonas aeruginosa Phage K8 and Its Genomic Analysis
Source: Front Microbiol. 2016 Mar 2;7:252. doi: 10.3389/fmicb.2016.00252 (PMC4773840; doi:10.3389/fmicb.2016.00252)

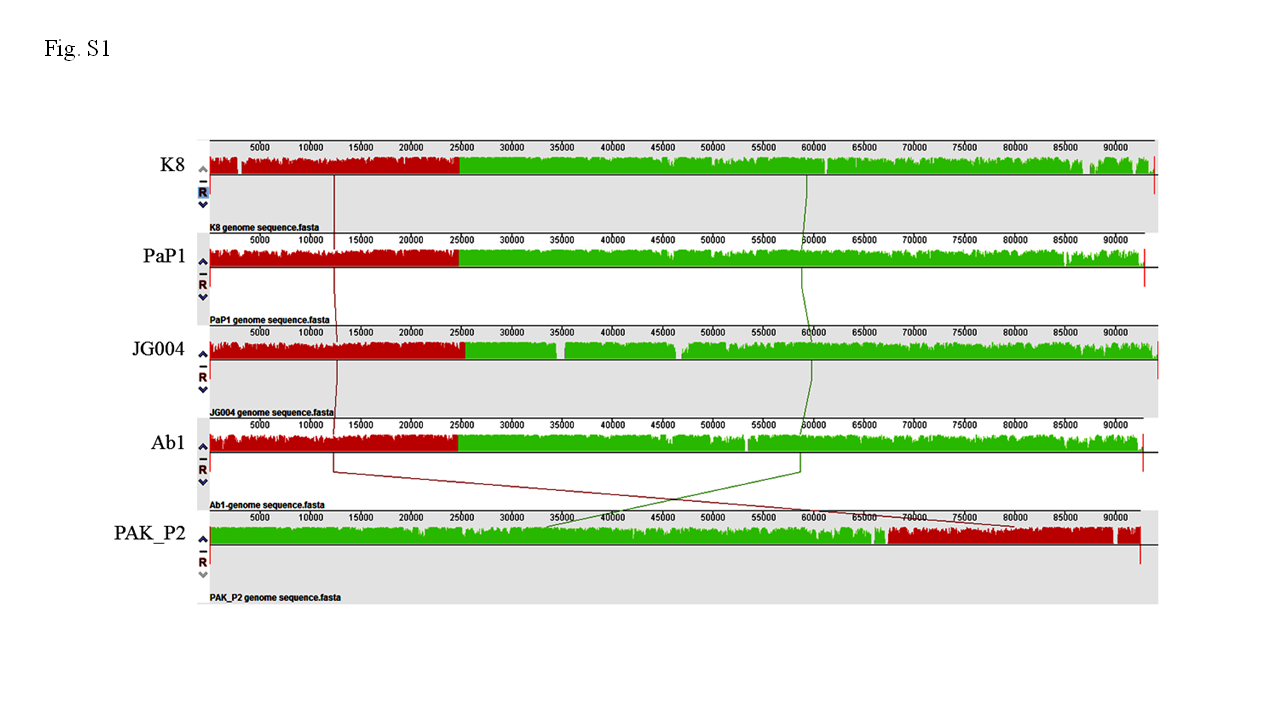

Supplement: FIGURE S1 — Comparative genomic analyses of Pseudomonas aeruginosa phages. Ab1: vB_PaeM_C2-10_Ab1. The coordinate rulers display the size of the corresponding genomes. The height of the red and green ribbons is correlated with the level of similarities between every two genomes. Different colors indicate the inconsistent genomic organizations among the phages. [file Image_1.TIF]
