# Supplementary material for: Genetic Evidence for O-Specific Antigen as Receptor of Pseudomonas aeruginosa Phage K8 and Its Genomic Analysis
Source: Front Microbiol. 2016 Mar 2;7:252. doi: 10.3389/fmicb.2016.00252 (PMC4773840; doi:10.3389/fmicb.2016.00252)
Supplement: Supplementary file 2 [file Table_1.DOC]

Table S1 Inserted genes in the phage-resistant mutants

| Strain | Inserted gene (bp) | Insertions site | Homolog | Protein | Pathway |
| --- | --- | --- | --- | --- | --- |
| SK21 | *Y880_RS05480* (1254) | between 70 bp and 71 bp | *PALES_16971* | Wzy | O-antigen synthesis |
| SK24 |  | between 94 bp and 95 bp |  |  |  |
| SK41 |  | between 35 bp and 36 bp |  |  |  |
| SK73 |  | between 790 bp and 791 bp |  |  |  |
| SK75 |  | between 94 bp and 95 bp |  |  |  |
| SK88 |  | between 370 bp and 371 bp |  |  |  |
| SK92 |  | between 388 bp and 389 bp |  |  |  |
| SK2 | *Y880_RS04310* (963) | between 543 bp and 544 bp | *wbpV* | WbpV | OSA synthesis |
| SK23 |  | between 391 bp and 392 bp |  |  |  |
| SK16 |  | between 397 bp and 398 bp |  |  |  |
| SK91 |  | between 397 bp and 398 bp |  |  |  |
| SK28 | *Y880_RS04330* (1047) | between 19 bp and 20 bp | *wbpR* | WbpR | OSA synthesis |
| SK45 |  | between 53 bp and 54 bp |  |  |  |
| SK5 | *PAK_02038* (1269) | between 723 bp and 724 bp | *wbpO* | WbpO | OSA synthesis |
| SK15 |  | between 723 bp and 724 bp |  |  |  |
| SK98 | *Y880_RS14550* (957) | between 338 bp and 339 bp | *ssg* | Ssg | O-antigen synthesis |
