# Supplementary material for: Genetic Evidence for O-Specific Antigen as Receptor of Pseudomonas aeruginosa Phage K8 and Its Genomic Analysis
Source: Front Microbiol. 2016 Mar 2;7:252. doi: 10.3389/fmicb.2016.00252 (PMC4773840; doi:10.3389/fmicb.2016.00252)
Supplement: Supplementary file 3 [file Table_2.DOC]

Table S2 Annotation of phage K8 genome

| Gene | Start | Stop | Strand | Homolog | Ident (%) | MW (kD) | Functions |
| --- | --- | --- | --- | --- | --- | --- | --- |
| 001 | 22 | 159 | Minus | PaP1_gp156 | 100 | 5.01 | hypothetical protein |
| 002 | 344 | 946 | Plus | PaP1_gp157 | 97.00 | 22.94 | hypothetical protein |
| 003 | 1468 | 1617 | Plus | PJG4_003 | 98.00 | 5.00 | hypothetical protein |
| 004 | 1681 | 1947 | Minus | PAK_P200126c | 100 | 10.59 | hypothetical protein |
| 005 | 1947 | 2351 | Minus | PAK_P200127c | 99.25 | 15.34 | hypothetical protein |
| 006 | 2341 | 2757 | Minus | PJG4_006 | 98.55 | 16.11 | hypothetical protein |
| 007 | 2754 | 3158 | Minus | ORF 07 | 100 | 15.99 | hypothetical protein |
| 008 | 3201 | 3863 | Minus | ORF 08 | 98.64 | 26.21 | hypothetical protein |
| 009 | 3866 | 4144 | Minus | PaP1_gp006 | 96.70 | 10.69 | hypothetical protein |
| 010 | 4132 | 4443 | Minus | PAK_P400130c | 98.06 | 12.03 | hypothetical protein |
| 011 | 4443 | 4805 | Minus | PAK_P100138c | 96.67 | 14.07 | hypothetical protein |
| 012 | 4862 | 5077 | Minus | PAK_P100139c | 91.55 | 8.28 | hypothetical protein |
| 013 | 5074 | 5415 | Minus | PJG4_011 | 97.35 | 12.59 | hypothetical protein |
| 014 | 5417 | 5920 | Minus | BN405_2-10_Ab1_orf_12 | 98.21 | 19.31 | hypothetical protein |
| 015 | 5907 | 6284 | Minus | PAK_P200137c | 100 | 15.03 | hypothetical protein |
| 016 | 6304 | 6924 | Minus | PJG4_014 | 94.55 | 23.07 | hypothetical protein |
| 017 | 6882 | 7199 | Minus | PaP1_gp014 | 76.19 | 12.08 | hypothetical protein |
| 018 | 7201 | 7350 | Minus | PaP1_gp015 | 97.96 | 5.74 | hypothetical protein |
| 019 | 7362 | 7841 | Minus | PAK_P100146c | 99.37 | 17.78 | hypothetical protein |
| 020 | 7838 | 8035 | Minus | PJG4_017 | 98.46 | 7.78 | hypothetical protein |
| 021 | 8047 | 9735 | Minus | PAK_P100148c | 99.17 | 63.06 | NPR transferase |
| 022 | 9688 | 9834 | Plus | — | — | 5.24 | — |
| 023 | 9792 | 10010 | Minus | BN405_2-10_Ab1_orf_18 | 98.61 | 8.77 | hypothetical protein |
| 024 | 10007 | 10873 | Minus | PaP1_gp020 | 100 | 31.88 | PRP synthetase |
| 025 | 10083 | 11299 | Minus | PaP1_gp021 | 100 | 15.86 | putative ATPase |
| 026 | 11310 | 12227 | Minus | PAK_P200147c | 98.69 | 35.02 | putative RNA ligase |
| 027 | 12239 | 12646 | Minus | PAK_P200148c | 98.52 | 15.22 | hypothetical protein |
| 028 | 12643 | 12918 | Minus | PJG4_024 | 95.60 | 10.33 | hypothetical protein |
| 029 | 12920 | 13156 | Minus | PAK_P100155c | 91.03 | 8.71 | hypothetical protein |
| 030 | 13168 | 13722 | Minus | PAK_P4 | 100 | 22.03 | putative phosphoesterase |
| 031 | 13722 | 14153 | Minus | PJG4_027 | 95.11 | 17.16 | hypothetical protein |
| 032 | 14143 | 14703 | Minus | PJG4_028 | 99.46 | 21.14 | putative phosphohydrolase |
| 033 | 14705 | 15265 | Minus | PJG4_029 | 97.85 | 21.50 | putative cell wall hydrolase |
| 034 | 15323 | 15787 | Minus | PaP1_gp030 | 100 | 17.35 | hypothetical protein |
| 035 | 15800 | 17008 | Minus | PAK_P400155c | 100 | 46.30 | DNA ligase |
| 036 | 17005 | 17421 | Minus | PaP1_gp032 | 100 | 15.46 | putative dCMP deaminase |
| 037 | 17424 | 17660 | Minus | PaP1_gp033 | 98.72 | 8.90 | hypothetical protein |
| 038 | 17670 | 17882 | Minus | PJG4_034 | 100 | 7.47 | hypothetical protein |
| 039 | 17879 | 18148 | Minus | PJG4_035 | 100 | 10.00 | hypothetical protein |
| 040 | 18157 | 18507 | Minus | PAK_P100167c | 100 | 13.23 | hypothetical protein |
| 041 | 18488 | 18766 | Minus | PJG4_038 | 97.83 | 10.56 | hypothetical protein |
| 042 | 18763 | 19041 | Minus | PAK_P100169c | 100 | 10.44 | hypothetical protein |
| 043 | 19076 | 19261 | Minus | PaP1_gp038 | 100 | 7.12 | hypothetical protein |
| 044 | 19262 | 19504 | Minus | P3_CHA0133 | 94.55 | 9.25 | hypothetical protein |
| 045 | 19501 | 19686 | Minus | PJG4_041 | 98.36 | 7.16 | hypothetical protein |
| 046 | 19747 | 20295 | Minus | PaP1_gp041 | 97.25 | 20.45 | protease subunit |
| 047 | 20343 | 20699 | Minus | PJG4_043 | 100 | 13.01 | hypothetical protein |
| 048 | 20696 | 21163 | Minus | PJG4_044 | 99.35 | 18.09 | hypothetical protein |
| 049 | 21968 | 22291 | Plus | PJG4_045 | 100 | 12.07 | hypothetical protein |
| 050 | 23173 | 23280 | Plus | — | — | 3.86 | — |
| 051 | 24182 | 24580 | Minus | PAK_P100181c | 100 | 15.25 | hypothetical protein |
| 052 | 24785 | 26305 | Plus | PJG4_059 | 100 | 57.33 | terminase large subunit |
| 053 | 26318 | 27757 | Plus | PaP1_gp047 | 100 | 54.38 | phage conserved protein |
| 054 | 27767 | 28237 | Plus | PaP1_gp048 | 100 | 17.24 | DNA methyltransferase |
| 055 | 28234 | 29151 | Plus | PaP1_gp049 | 100 | 33.11 | hypothetical protein |
| 056 | 29179 | 29589 | Plus | PAK_P100005 | 100 | 14.89 | hypothetical protein |
| 057 | 29633 | 30667 | Plus | PaP1_gp051 | 100 | 39.50 | major capsid protein |
| 058 | 30717 | 31193 | Plus | PAK_P100007 | 100 | 18.17 | hypothetical protein |
| 059 | 31231 | 31653 | Plus | PJG4_066 | 100 | 15.67 | putative RNA polymerase |
| 060 | 31644 | 32024 | Plus | PJG4_067 | 100 | 14.39 | hypothetical protein |
| 061 | 32021 | 32584 | Plus | PJG4_068 | 100 | 21.33 | hypothetical protein |
| 062 | 32597 | 33883 | Plus | PaP1_gp56 | 98.60 | 46.46 | putative structural protein |
| 063 | 33914 | 34438 | Plus | PaP1_gp57 | 100 | 19.02 | putative structural protein |
| 064 | 34513 | 35013 | Plus | BN405_2-10_Ab1_orf_59 | 100 | 18.30 | hypothetical protein |
| 065 | 35013 | 35492 | Plus | PaP1_gp59 | 100 | 17.76 | putative structural protein |
| 066 | 35506 | 35877 | Plus | PaP1_gp60 | 100 | 13.63 | putative structural protein |
| 067 | 35985 | 36137 | Plus | PAK_P200016 | 100 | 5.61 | hypothetical protein |
| 068 | 36134 | 38500 | Plus | PaP1_gp061 | 100 | 86.11 | putative TMP |
| 069 | 38497 | 39258 | Plus | PaP1_gp062 | 100 | 28.62 | hypothetical protein |
| 070 | 39264 | 39620 | Plus | PaP1_gp063 | 100 | 14.02 | hypothetical protein |
| 071 | 39617 | 40534 | Plus | PAK_P400021 | 100 | 33.96 | hypothetical protein |
| 072 | 40531 | 41271 | Plus | PaP1_gp065 | 100 | 26.76 | putative baseplate protein |
| 073 | 41282 | 41653 | Plus | BN405_2-10_Ab1_orf_68 | 100 | 14.21 | hypothetical protein |
| 074 | 41655 | 43118 | Plus | PaP1_gp067 | 100 | 52.51 | base plate related protein |
| 075 | 43137 | 43868 | Plus | PaP1_gp068 | 100 | 26.73 | hypothetical protein |
| 076 | 43879 | 45891 | Plus | PAK_P400026 | 100 | 70.19 | putative tail fiber protein |
| 077 | 45928 | 46305 | Plus | PAK_P400027 | 100 | 14.91 | hypothetical protein |
| 078 | 46319 | 47818 | Plus | PaP1_gp71 | 99.27 | 53.34 | putative tail fiber protein |
| 079 | 47835 | 48395 | Plus | PaP1_gp072 | 100 | 21.01 | endolysin |
| 080 | 48413 | 48652 | Plus | PaP1_gp073 | 100 | 8.52 | hypothetical protein |
| 081 | 48639 | 49082 | Plus | BN405_2-10_Ab1_orf_76 | 100 | 15.92 | hypothetical protein |
| 082 | 49093 | 49224 | Plus | PAK_P200031 | 100 | 4.87 | hypothetical protein |
| 083 | 49214 | 49519 | Plus | PAK_P100032 | 100 | 11.44 | hypothetical protein |
| 084 | 49555 | 49869 | Plus | PAK_P200033 | 100 | 12.38 | hypothetical protein |
| 085 | 49907 | 50218 | Minus | PaP1_gp076 | 93.21 | 11.63 | hypothetical protein |
| 086 | 50230 | 50550 | Minus | PJG4_093 | 100 | 12.19 | hypothetical protein |
| 087 | 50568 | 51380 | Minus | PAK_P100036c | 98.52 | 30.89 | pyrophosphatase |
| 088 | 51373 | 51531 | Minus | PaP1_gp078 | 100 | 5.79 | hypothetical protein |
| 089 | 51534 | 52676 | Minus | PaP1_gp079 | 91.84 | 42.92 | RNA ligase |
| 090 | 52708 | 52941 | Minus | PJG4_098 | 100 | 8.54 | hypothetical protein |
| 091 | 53382 | 53579 | Plus | PJG4_101 | 96.92 | 7.45 | hypothetical protein |
| 092 | 53582 | 54067 | Plus | PAK_P200042 | 99.38 | 19.26 | hypothetical protein |
| 093 | 54095 | 54343 | Plus | BN405_2-10_Ab1_orf_87 | 57.65 | 9.27 | hypothetical protein |
| 094 | 54366 | 54734 | Plus | PAK_P200042 | 99.38 | 14.08 | hypothetical protein |
| 095 | 54731 | 55387 | Plus | PaP1_gp083 | 100 | 24.21 | hypothetical protein |
| 096 | 55374 | 55538 | Plus | BN405_2-10_Ab1_orf_90 | 100 | 6.43 | hypothetical protein |
| 097 | 55541 | 55843 | Plus | PaP1_gp085 | 100 | 11.94 | hypothetical protein |
| 098 | 55844 | 56266 | Plus | PAK_P100048 | 100 | 16.56 | hypothetical protein |
| 099 | 56275 | 56391 | Plus | PAK_P200048.1 | 100 | 4.39 | hypothetical protein |
| 100 | 56384 | 56512 | Plus | PAK_P400049 | 100 | 5.13 | hypothetical protein |
| 101 | 56499 | 56690 | Plus | PAK_P400050 | 100 | 7.14 | hypothetical protein |
| 102 | 56700 | 56945 | Plus | PAK_P400051 | 100 | 9.22 | hypothetical protein |
| 103 | 56942 | 57127 | Plus | PJG4_109 | 100 | 7.22 | hypothetical protein |
| 104 | 57181 | 59043 | Plus | PJG4_110 | 100 | 70.86 | DNA primase/helicase |
| 105 | 59104 | 61113 | Plus | PAK_P100055 | 99.55 | 77.97 | DNA polymerase B family |
| 106 | 61398 | 62087 | Plus | PJG4_111 | 99.56 | 7.67 | DNA polymerase A family |
| 107 | 62177 | 62575 | Plus | PAK_P400055 | 100 | 7.92 | hypothetical protein |
| 108 | 62604 | 62771 | Plus | PaP1_gp092 | 100 | 6.15 | hypothetical protein |
| 109 | 62773 | 63483 | Plus | PaP1_gp093 | 100 | 26.34 | hypothetical protein |
| 110 | 63585 | 64589 | Plus | PJG4_114 | 100 | 37.24 | hypothetical protein |
| 111 | 64659 | 64892 | Plus | PJG4_115 | 100 | 8.42 | hypothetical protein |
| 112 | 64902 | 65123 | Plus | PJG4_116 | 100 | 8.25 | hypothetical protein |
| 113 | 65165 | 66217 | Plus | PJG4_117 | 100 | 40.12 | putative exodeoxyribonuclease |
| 114 | 66214 | 66777 | Plus | PaP1_gp098 | 100 | 21.66 | phage protein |
| 115 | 66774 | 67172 | Plus | LU11_gp114 | 40.77 | 15.25 | putative endolysin |
| 116 | 67169 | 67399 | Plus | PAK_P200064 | 75.00 | 8.71 | hypothetical protein |
| 117 | 67396 | 67833 | Plus | PAK_P100066 | 93.11 | 16.61 | hypothetical protein |
| 118 | 67830 | 67997 | Plus | PAK_P100067 | 100 | 6.47 | hypothetical protein |
| 119 | 67999 | 68181 | Plus | PaP1_gp103 | 100 | 7.07 | hypothetical protein |
| 120 | 68178 | 68957 | Plus | PaP1_gp104 | 100 | 29.43 | constituent protein |
| 121 | 68954 | 69136 | Plus | PaP1_gp105 | 100 | 6.99 | hypothetical protein |
| 122 | 69156 | 69365 | Plus | PAK_P100071 | 100 | 7.51 | hypothetical protein |
| 123 | 69385 | 69720 | Plus | PaP1_gp107 | 100 | 12.48 | hypothetical protein |
| 124 | 69724 | 69939 | Plus | PaP1_gp108 | 100 | 7.91 | hypothetical protein |
| 125 | 69932 | 70882 | Plus | PaP1_gp109 | 100 | 35.35 | hypothetical protein |
| 126 | 70918 | 71034 | Plus | PAK_P100075 | 97.37 | 4.63 | hypothetical protein |
| 127 | 71089 | 72024 | Plus | PaP1_gp110 | 100 | 37.03 | thymidylate synthase |
| 128 | 72053 | 72397 | Plus | PaP1_gp111 | 100 | 13.22 | hypothetical protein |
| 129 | 72414 | 73460 | Plus | BN405_2-10_Ab1_orf_117 | 100 | 40.47 | RDR beta subunit |
| 130 | 73453 | 75198 | Plus | PaP1_gp113 | 99.31 | 66.88 | RDR alpha chain |
| 131 | 75273 | 75416 | Plus | PAK_P100080 | 97.87 | 5.48 | hypothetical protein |
| 132 | 75413 | 75649 | Plus | PAK_P100081 | 98.72 | 9.56 | hypothetical protein |
| 133 | 75649 | 75870 | Plus | PAK_P100082 | 100 | 8.41 | hypothetical protein |
| 134 | 75891 | 76130 | Plus | PAK_P100083 | 100 | 9.22 | hypothetical protein |
| 135 | 76127 | 76402 | Plus | PaP1_gp117 | 100 | 10.5 | hypothetical protein |
| 136 | 76404 | 76781 | Plus | PaP1_gp118 | 100 | 13.76 | hypothetical protein |
| 137 | 76787 | 76972 | Plus | PaP1_gp119 | 100 | 6.97 | hypothetical protein |
| 138 | 77018 | 77257 | Plus | PAK_P100087 | 100 | 9.09 | hypothetical protein |
| 139 | 77277 | 77780 | Plus | PaP1_gp121 | 99.40 | 18.44 | hypothetical protein |
| 140 | 77790 | 77984 | Plus | PAK_P100089 | 100 | 7.32 | hypothetical protein |
| 141 | 77986 | 78216 | Plus | PAK_P100090 | 100 | 8.73 | hypothetical protein |
| 142 | 78289 | 78489 | Plus | PaP1_gp124 | 100 | 8.21 | hypothetical protein |
| 143 | 78647 | 79633 | Plus | PAK_P100092 | 99.70 | 37.8 | hypothetical protein |
| 144 | 79856 | 80029 | Plus | PAK_P100093 | 100 | 6.93 | hypothetical protein |
| 145 | 80654 | 81127 | Plus | PAK_P100094 | 100 | 18.17 | hypothetical protein |
| 146 | 81215 | 81484 | Plus | PAK_P100095 | 98.88 | 10.5 | hypothetical protein |
| 147 | 81497 | 81640 | Plus | PaP1_gp128 | 97.78 | 5.44 | hypothetical protein |
| 148 | 81640 | 81927 | Plus | PaP1_gp129 | 100 | 10.59 | hypothetical protein |
| 149 | 81939 | 82172 | Plus | PAK_P400095 | 97.41 | 8.44 | hypothetical protein |
| 150 | 82243 | 82371 | Plus | PAK_P100099 | 100 | 4.86 | hypothetical protein |
| 151 | 82371 | 82679 | Plus | PAK_P200097 | 100 | 11.42 | hypothetical protein |
| 152 | 82753 | 82899 | Plus | PaP1_gp133 | 100 | 5.58 | hypothetical protein |
| 153 | 82994 | 83143 | Plus | PAK_P200099 | 100 | 5.53 | hypothetical protein |
| 154 | 83136 | 83519 | Plus | PaP1_gp134 | 100 | 15.22 | hypothetical protein |
| 155 | 83595 | 84266 | Plus | PJG4_154 | 99.10 | 24.47 | hypothetical protein |
| 156 | 84271 | 84159 | Plus | BN405_2-10_Ab1_orf_139 | 98.21 | 12.56 | hypothetical protein |
| 157 | 84609 | 84962 | Plus | PAK_P400102 | 100 | 12.97 | hypothetical protein |
| 158 | 85013 | 85153 | Plus | PAK_P400103 | 100 | 4.33 | hypothetical protein |
| 159 | 85226 | 85420 | Plus | PJG4_158 | 96.88 | 6.95 | hypothetical protein |
| 160 | 85436 | 85732 | Plus | PAK_P200107 | 83.67 | 11.38 | hypothetical protein |
| 161 | 85729 | 85953 | Plus | PaP1_gp140 | 95.95 | 8.45 | hypothetical protein |
| 162 | 85986 | 86252 | Plus | PJG4_161 | 96.59 | 10.01 | hypothetical protein |
| 163 | 86249 | 86641 | Plus | PaP1_gp142 | 97.60 | 15.09 | hypothetical protein |
| 164 | 86746 | 87000 | Plus | — | — | 10.23 | — |
| 165 | 87078 | 87434 | Plus | — | — | 13.43 | — |
| 166 | 87477 | 88058 | Plus | PaP1_gp143 | 64.06 | 21.76 | hypothetical protein |
| 167 | 88193 | 88384 | Plus | PAK_P100116 | 64.62 | 7.23 | hypothetical protein |
| 168 | 88420 | 88899 | Plus | PJG4_166 | 98.11 | 18.03 | hypothetical protein |
| 169 | 88983 | 89498 | Plus | PAK_P200114 | 99.42 | 19.59 | hypothetical protein |
| 170 | 89573 | 90100 | Plus | PAK_P400114 | 100 | 19.56 | hypothetical protein |
| 171 | 90169 | 90381 | Plus | PJG4_168 | 100 | 7.88 | hypothetical protein |
| 172 | 90409 | 90573 | Plus | PAK_P100120 | 94.44 | 6.22 | hypothetical protein |
| 173 | 90628 | 91080 | Plus | BN405_2-10_Ab1_orf_153 | 99.33 | 17.4 | hypothetical protein |
| 174 | 91179 | 91325 | Plus | PaP1_gp151 | 97.92 | 5.49 | hypothetical protein |
| 175 | 91322 | 91621 | Plus | PAK_P400118 | 96.81 | 11.52 | hypothetical protein |
| 176 | 91694 | 91948 | Plus | PAK_P500107 | 100 | 9.33 | hypothetical protein |
| 177 | 91998 | 92258 | Plus | PAK_P200122 | 98.84 | 9.59 | hypothetical protein |
| 178 | 92713 | 92850 | Minus | PaP1_gp156 | 100 | 5.01 | hypothetical protein |
| 179 | 93035 | 93637 | Plus | PaP1_gp157 | 97.00 | 22.94 | hypothetical protein |

NPR: nicotinamide phosphoribosyl; PRP: phosphoribosyl pyrophosphate; RDR: ribonucleotide diphosphate reductase; TMP: tape measure protein; ‘—’ represents the encoded protein is not homologous to any phage proteins. ORF 07: ORF 07 of *Pseudomonas* phage vB_PaeM_C2-10_Ab02. ORF 08: ORF 08 of *Pseudomonas* phage vB_PaeM_C2-10_Ab02.
